# Shading Effects on Leaf Gas Exchange, Leaf Pigments and Secondary Metabolites of *Polygonum minus* Huds., an Aromatic Medicinal Herb

**DOI:** 10.3390/plants10030608

**Published:** 2021-03-23

**Authors:** Fairuz Fatini Mohd Yusof, Jamilah Syafawati Yaacob, Normaniza Osman, Mohd Hafiz Ibrahim, Wan Abd Al Qadr Imad Wan-Mohtar, Zulkarami Berahim, Nurul Amalina Mohd Zain

**Affiliations:** 1 Faculty of Science, Institute of Biological Sciences, Universiti Malaya, Kuala Lumpur 50603, Malaysia; fatinifairuz@siswa.um.edu.my (F.F.M.Y.); jamilahsyafawati@um.edu.my (J.S.Y.); normaniza@um.edu.my (N.O.); 2 Department of Biology, Faculty of Science, Universiti Putra Malaysia, Serdang 43400, Malaysia; mhafiz_ibrahim@upm.edu.my; 3 Functional Omics and Bioprocess Development Laboratory, Faculty of Science, Institute of Biological Sciences, Universiti Malaya, Kuala Lumpur 50603, Malaysia; qadyr@um.edu.my; 4 Laboratory of Climate-Smart Food Crop Production, Institute of Tropical Agriculture and Food Security, Universiti Putra Malaysia, Serdang 43400, Malaysia; zulkerami@upm.edu.my

**Keywords:** *Polygonum minus*, shading levels, growth, leaf gas exchange, leaf pigments, secondary metabolites

## Abstract

The growing demand for high value aromatic herb *Polygonum minus*-based products have increased in recent years, for its antioxidant, anticancer, antimicrobial, and anti-inflammatory potentials. Although few reports have indicated the chemical profiles and antioxidative effects of *Polygonum minus*, no study has been conducted to assess the benefits of micro-environmental manipulation (different shading levels) on the growth, leaf gas exchange and secondary metabolites in *Polygonum minus*. Therefore, two shading levels (50%:T2 and 70%:T3) and one absolute control (0%:T1) were studied under eight weeks and 16 weeks of exposures on *Polygonum minus* after two weeks. It was found that *P. minus* under T2 obtained the highest photosynthesis rate (14.892 µmol CO_2_ m^−2^ s^−1^), followed by T3 = T1. The increase in photosynthesis rate was contributed by the enhancement of the leaf pigments content (chlorophyll a and chlorophyll b). This was shown by the positive significant correlations observed between photosynthesis rate with chlorophyll a (r^2^ = 0.536; *p* ≤ 0.05) and chlorophyll b (r^2^ = 0.540; *p* ≤ 0.05). As the shading levels and time interval increased, the production of total anthocyanin content (TAC) and antioxidant properties of Ferric Reducing Antioxidant Power (FRAP) and 2,2-Diphenyl-1-picrylhydrazyl (DPPH) also increased. The total phenolic content (TPC) and total flavonoid content (TFC) were also significantly enhanced under T2 and T3. The current study suggested that *P.minus* induce the production of more leaf pigments and secondary metabolites as their special adaptation mechanism under low light condition. Although the biomass was affected under low light, the purpose of conducting the study to boost the bioactive properties in *Polygonum minus* has been fulfilled by 50% shading under 16 weeks’ exposure.

## 1. Introduction

*Polygonum minus* Huds (Family: Polygonaceae), which is commonly known as kesum, is an aromatic herbaceous plant originally located from Southeast Asian countries, such as Malaysia, Thailand, Vietnam, and Indonesia [1]. *P. minus* is frequently used by the locals in Malaysia as spices and flavouring agents in the delicate local cuisine due to its distinctive and lemony aroma. It has also been traditionally used to treat digestive disorders, dandruff problems and body [2]. Previous research identified that decanal (24.36%) and dodecanal (48.18%) are the two dominant aldehydes that contribute to the flavour of kesum [3]. High levels of aliphatic aldehydes (72.54%) produced in *P. minus* recognised it as an essential oil-producing crop in the Herbal Product Blueprint by the Malaysian government [4]. It also contains a high level of flavonoid and phenolic compounds, which contributes to its notable bioactive properties, such as antioxidant, antimicrobial, antifungal, antiviral, and antiulcer activities [5]. *P. minus* has also been reported to exhibit significant reactive oxygen species (ROS) scavenging activities due to its high antioxidant content, and it can be considered to be as one of natural medicinal resources [6]. *P. minus* was also reported to be able to improve cognitive function and exhibit neuroprotective effect [7]. Currently, few *P. minus* or kesum-based products have been commercialised, for its antioxidant, anticancer, antimicrobial, and anti-inflammatory potentials [2,5]. The potential of *P. minus* as an anticancer agent was recognised by the display of anti-proliferative activity and apoptosis induction in *P. minus* on selected cancer and normal cell lines [8]. Another study has succeeded in isolating a new compound from *P. minus* stems, named Polygonumins A, which was reported to be able to inhibit cancer cells without affecting the normal cells, and have the potential in treating leukaemia [9]. Therefore, the demand of this herb increases due to the high compound of natural antioxidants.

Light intensity is known to regulate not only plant growth and development, but also the biosynthesis of secondary metabolites [10,11]. In general, plants living in a full light intensity exhibit different photosynthetic and leaf characteristics than those that are living under the shade. Studies have been carried out to examine environmental stress such as temperature and light on growth and development of several plant species. A study on *Orthosiphon stamineus* showed that low light helped the plant to produce higher biomass when compared to high light intensity [12]. A similar result was also obtained in *Epimedium pseudowushanense* exposed to light intensity level of 90.9 ± 2.5 µmol m^−2^ s^−1^ [13]. However, these results were in contrast to that obtained in young tomato plant, which showed that the highest light intensity resulted in the highest accumulation of biomass [14]. It was also reported that low light negatively affects stomatal conductance and it resulted in the enhanced concentration of intercellular CO_2_ in rice leaves [15]. Meanwhile, another study by Zhu et al., 2017 [16] showed that the chlorophyll b pigments, intracellular CO₂ content, and stomatal conductance of the plant *Brassica campestris* increased under shaded treatments. This indicates that the effects of shadings or light intensity vary with different plant species. Different irradiance levels exposed to plants will also influence and affect the production and accumulation of secondary metabolites. Ghamzadeh et al., 2010 [17] reported that light can stimulate the production of secondary metabolites, such as gingerol and zingiberine in ginger, *Zingiber officinale*. Meanwhile, another study conducted by Ibrahim et al., 2014 [18] on the plant *Labisia pumila* showed an increase in the production of secondary metabolites at low light intensity level, along with a high level of CO_2_.

Currently, the knowledge of different environmental effects on *P. minus* is still limited. Although various reports can be found on the chemical profiles and antioxidative effects of *Polygonum minus* [3,5], no study was conducted for the effects of shading on the production of secondary metabolites and antioxidant activity in *Polygonum minus*. A sufficient understanding on the influence of shading towards the growth, leaf gas exchange, and the production of secondary metabolites in *P. minus* are very important for successful cultivation and optimisation in order to utilise this plant’s benefit efficiently. This current study was conducted with the objective to examine the effects of different light intensity under different shading levels on the growth, leaf gas exchange, and the accumulation of secondary metabolites of *P. minus.*

## 2. Results

### 2.1. Plant Growth

**Figure 1:**
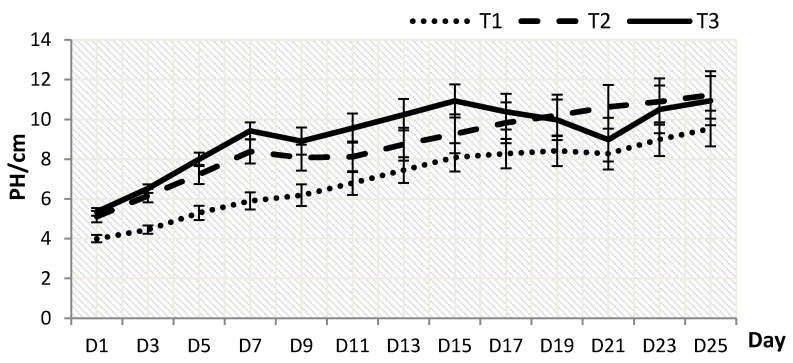
Effect of shading levels on plant height, PH (Data are means of treatments, *N* = 96; Rep = 4; Control, T1 = 0% Shaded; T2 = 50% Shading and T3 = 70% Shading; Small bars represent standard error).

**Figure 2:**
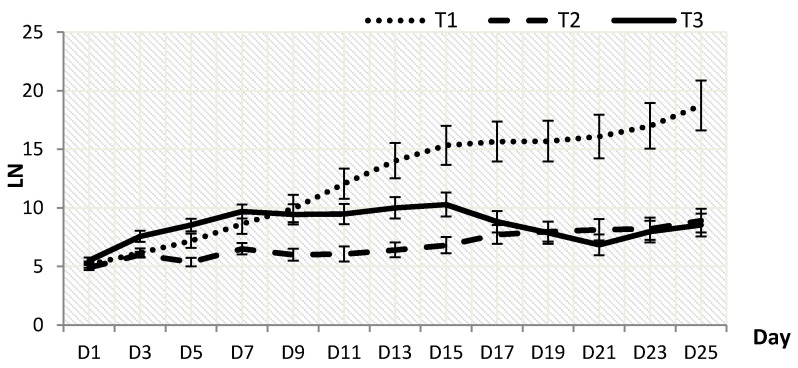
Effect of shading levels on leaves number, LN (Data are means of treatments, *N* = 96; Rep = 4; Control, T1 = 0% Shading; T2 = 50% Shading and T3 = 70% Shading; Small bars represent standard error).

Plant height (PH) and leaves number (LN) were significantly influenced by the different shading levels. *P. minus* grown under T2 grew the tallest with PH of 11.23 ± 1.197 cm on the 25th day, followed by T3 (10.94 ± 1.234 cm) and T1 (9.54 ± 0.895 cm) (Figure 1). Data analysis shows that T3 resulted in the increment of plant height, but the results dropped after the 17th day and they showed the lowest drop on the 21st day, due to wilting symptoms that were exhibited by a few plants. This sudden drop trend on the 21st day can also be seen in LN (Figure 2).

T1 resulted in the highest LN (18.75 ± 2.133), which was 52.5% higher when compared to T2 (8.91 ± 1.011) and 54.5% more as compared to T3 (8.53 ± 0.979) recorded on the 25th day (Figure 2). The decreasing trend under T3 was observed to occur from the 17th day until the 21st day before re-increasing in number on the 23rd day, possibly due to the low light penetration that caused LN reduction in *P. minus*. Meanwhile, a constant increasing trend was observed under T2 starting on the 9th day. PH had positively correlated with LN (r^2^ = 0.692, *p* ≤ 0.01).

### 2.2. Biomass Dry Weight and Root-Shoot Ratio(RSR)

**Figure 3:**
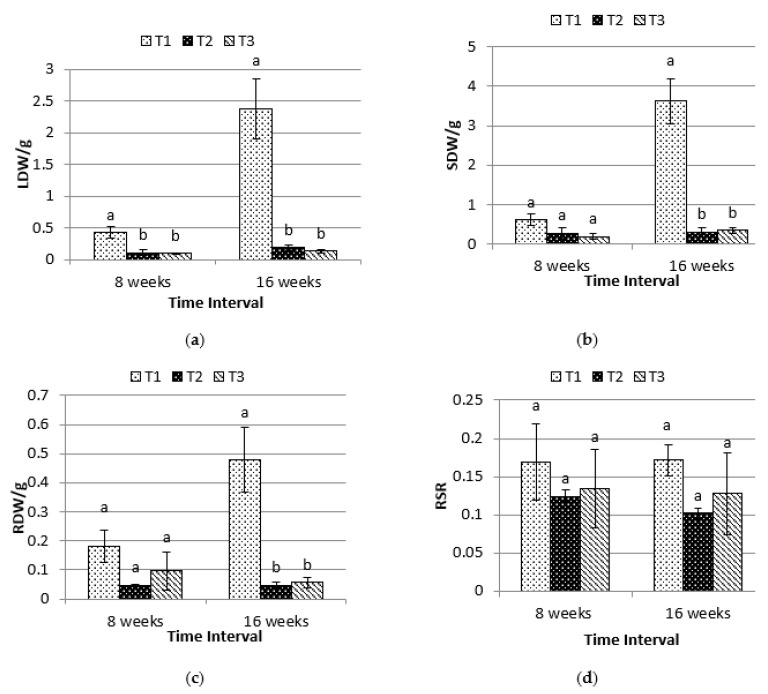
Effects of different shading levels (T1 = 0%; T2 = 50% and T3 = 70%) on (**a**) LDW, leaf dry weight; (**b**) SDW, shoot dry weight; (**c**) RDW, root dry weight; and, (**d**) RSR, root-shoot ratio under time interval of eight weeks and 16 weeks) (data are means of treatments, *N* = 48; Rep = 4; Control, T1 = 0% Shading; T2 = 50% Shading and T3 = 70% Shading; Small bars represent standard error). Means with different letters on top of each bar are significantly different at *p* ≤ 0.05.

Leaf dry weight (LDW), shoot dry weight (SDW), and root dry weight (RDW) that were harvested under time interval of 16 weeks were significantly influenced by different shading levels (*p* ≤ 0.05), while there are no significant differences among SDW and RDW under time interval of eight weeks excluding LDW. Under eight weeks, the lowest LDW found on T3 and T2. Figure 3a,b show control, T1 achieved the highest LDW and SDW in eight weeks and 16 weeks. On 16 weeks, the RDW obtained by T1 showed distinct root dry weight compared to T2 and T3. For RSR, there is no significant difference found on both time intervals. Pearson’s correlation shows that LDW has a strong correlation with SDW (r^2^ = 0.988, *p* ≤ 0.01) and RDW (r^2^ = 0.985, *p* ≤ 0.01).

### 2.3. Leaf Gas Exchange, Leaf Pigments and In-Situ Chlorophyll

The highest photosynthesis rate (A) was observed under T2 (14.89 ± 3.65 µ mol CO_2_ m^−2^ s^−1^), followed by T3 = T1. The same trend was observed for leaf temperature (Tleaves) with the highest value being recorded in plants under T2 (36.10 ± 1.061 °C) and the lowest temperature under T1 (33.81 ± 0.115 °C), as in Table 1. Other leaf gas exchange properties, such as the transpiration rate (E) and stomatal conductance (Gs), were also found to be significantly influenced by different shade levels (*p* ≤ 0.01). The highest E (0.3399 ± 0.0008 mmol H_2_O m^−2^ s^−1^) and Gs (0.007 ± 0.034 mol H_2_O m^−2^ s^−1^) were recorded under T2 > T1 = T3. Significant positive correlations (Table 2) were observed between A and leaf pigments, such as Chl a (r^2^ = 0.536, *p* ≤ 0.05), with Chl b (r^2^ = 0.540, *p* ≤ 0.05) and with Chl a + b (r^2^ = 0.546, *p* ≤ 0.05), which indicates that the increase in leaf pigments would increase the photosynthesis rate. There is also high positive correlation between E and Chl a (r^2^ = 0. 652, *p* ≤ 0.05), and Chl b (r^2^ = 0.553, *p* ≤ 0.05). Additionally, Gs were also shown to be positively correlated with Chl a (r^2^ = 0.62, *p* ≤ 0.05). These show that leaf gas exchange properties increase with the increase of leaves pigment content.

Leaf pigment contents were expressed as mg g^−1^ fresh weight (FW) were significantly influenced by different shading levels. The highest Chl a (5.251 ± 0.539 mg g^−1^ FW), Chl b (5.317 ± 0.785 mg g^−1^ FW), Chl a + b (10.568 ± 1.324 mg g^−1^ FW), and Car content (2.771 ± 0.369 mg g^−1^ FW) were obtained under T2, followed by T3 and the lowest under T1. However, there was non-significant difference between the treatments (*p* > 0.05) for chlorophyll a/b ratio. The highest in-situ chlorophyll content (SPAD) value was obtained under T2 (33.716 ± 1.198), followed by T3 (30.305 ± 0.916) and the lowest was under T1 (26.258 ± 0.822), where the values were significantly influenced by different shading levels. Table 1 shows all data. The rranspiration rate was positively correlated with Chl a (r^2^ = 0.652; *p* ≤ 0.05), Chl b (r^2^ = 0.553; *p* ≤ 0.05), and Chl a + b (r^2^ = 0.61; *p* ≤ 0.05) (Table 2).

### 2.4. Total Anthocyanin, Phenolic, and Flavonoid Content

**Figure 4:**
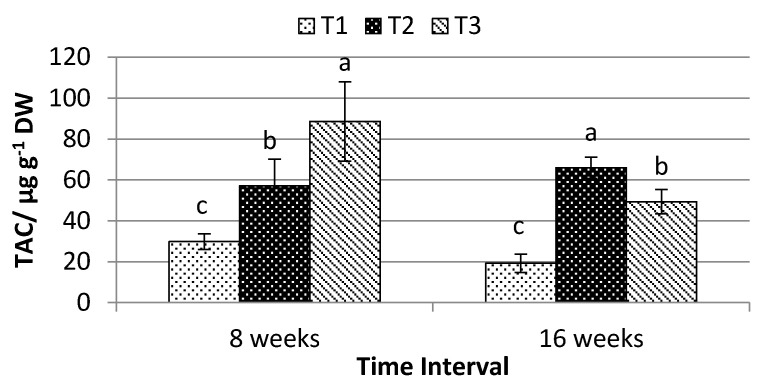
Effects of different shading levels on the total anthocyanin content of *P. minus* (data are means of treatments; *N* = 36; Rep = 3; Control, T1 = 0% Shading; T2 = 50% Shading; and, T3 = 70% Shading; DW = Dry Weight). Means with different letters on top of each bar are significantly different at *p* ≤ 0.05.

The TAC, TPC, and TFC of *P. minus* leaves were significantly influenced by different shading levels. Under time interval of eight weeks, T3 recorded the highest TAC (88.597 ± 19.368 μg g^−1^ dry weight (DW)), followed by T2 (57.055 ± 13.18 μg g^−1^ DW), and the lowest was obtained by T1 (29.90 ± 3.831 μg g^−1^ DW) (Figure 4). However, when the plants were exposed with longer treatment duration of 16 weeks, T2 was observed to yield the highest TAC (65.961 ± 5.205 μg g^−1^ DW), followed by T3 > T1 (Figure 4). Table 2 shows that there was a strong correlation observed between TAC with Car (r^2^ = 0.549, *p* ≤ 0.05).

**Figure 5:**
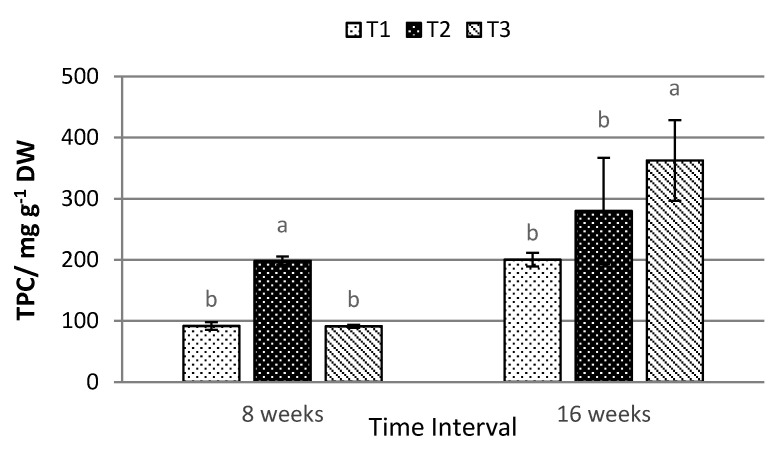
The effects of different shading levels on the total phenolic content of *P. minus* (data are means of treatments, *N* = 36; Rep = 3; Control, T1 = 0% Shading; T2 = 50% Shading and T3 = 70% Shading; DW = Dry Weight). Means with different letters on top of each bar are significantly different at *p* ≤ 0.05.

**Figure 6:**
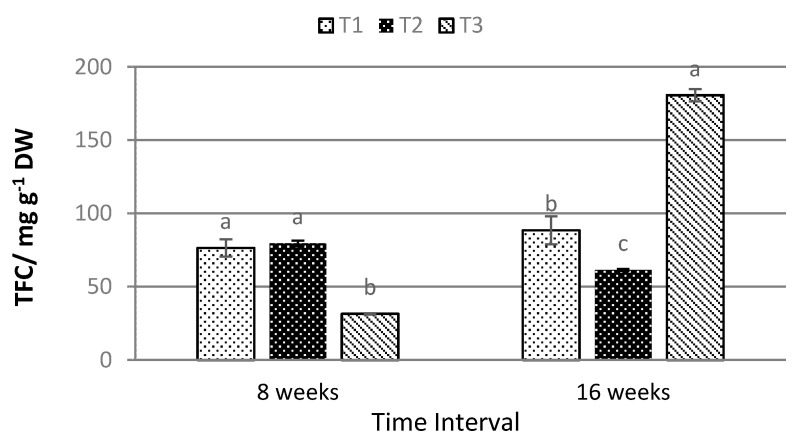
Effects of different shade levels on the total flavonoid content of *P. minus* (data are means of treatments, N = 36; Rep = 3; Control, T1 = 0% Shading; T2 = 50% Shading and T3 = 70% Shading; DW = Dry Weight). Means with different letters on top of each bar are significantly different at *p* ≤ 0.05.

Conversely, the highest TPC under eight weeks (198.286 ± 7.193 mg g^−1^ DW) was obtained under T2. Meanwhile, after 16 weeks of treatment exposure, the highest TPC was recorded under T3, with values of 362.323 ± 65.990 mg g^−1^ DW (Figure 5). The same trend was observed for TFC, where the highest flavonoid content was recorded under T3 (180.651 ± 4.219 mg g^−1^ DW) after 16 weeks of exposure (Figure 6).

Significant positive correlations can be seen between TPC and TFC with in-situ chlorophyll, SPAD (r^2^ = 0.744, *p* ≤ 0.01 and r^2^ = 0.644; *p* < 0.05 (Table 2), respectively. High significant correlations are also observed between TPC and TFC with Tleaves. with r^2^ values of 0.798, *p* ≤ 0.01 and 0.693, *p* ≤ 0.05.

### 2.5. Radical Scavenging Assays

The dry extract (DE) of the leaves from *P. minus* was also examined for its antioxidant capabilities. FRAP and DPPH radical scavenging assays were used to measure its antioxidant capacity. Table 3 presents the FRAP and the IC_50_ values. The interaction effect between shading treatments and time interval were both significantly different for the measurement of FRAP reducing power and IC_50_ values of DPPH. The highest FRAP reducing power was recorded under T2 of 16 weeks (2.616 ± 0.071 mg g^−1^ DE), followed by T1 eight week time interval (2.041 ± 0.004 mg g^−1^ DE) and the leaves extract under T3 have the lowest FRAP reducing power (1.457 ± 0.036 mg g^−1^ DE). The measurement for the highest FRAP reducing power was also parallel to the lowest concentration that is needed in order to inhibit 50% of the non-radical 1,1- diphenyl-2-picryl hydrazine. Leaves extract under T2 time interval of 16 weeks needed only 0.657 mg mL^−1^ of the extract to exhibit 50% inhibition, followed by T3 under time interval of 16 weeks. The highest IC_50_ were obtained under T1 time interval of 16 weeks, but the value was not significantly different when compared to the value of IC_50_ obtained under T1 time interval of eight weeks. Overall, the antioxidant capacities of *P. minus* were the highest under a T2 time interval of 16 weeks.

High positive significant correlation can be seen between FRAP with Chl a (r^2^ = 0.734; *p* < 0.01), Chl b (r^2^ = 0.598; *p* < 0.05), Car (r^2^ = 0.707; *p* < 0.01), and TAC (r^2^ = 0.859; *p* < 0.01). This means that, as the respective values increase, the FRAP reducing power will also increase. Meanwhile, DPPH was only negatively correlated with TAC (r^2^ = −0.666; *p* < 0.05). A significant negative relationship can also be seen between DPPH and FRAP (r^2^ = −0.59; *p* < 0.05). No significant correlation can be seen between both antioxidant assays with TPC and TFC. Table 4 records these correlation data.

## 3. Discussion

Based on overall data analysed, the different shading levels highly influenced the *P. minus* growth, leaf gas exchange properties, leaf pigments, and secondary metabolites. Referring to the growth data, PH and LN were significantly influenced by the difference shading levels, where the highest PH was obtained under T2 with 50% shading or 50% light penetration to the plant on 25th day (Figure 1). It was reported that the responses of plants towards high shading level (low light intensity) are many and varied between plant species, but rapid and higher growth, or growth towards light (phototropism), are common changes that could be observed [19,20,21]. Henceforth, the investment of plant in height will indirectly improve the access of plants towards light for better light absorbance and photosynthesis process [22], especially under shading condition. These findings are in agreement with the results that were reported by other studies on *Baccharis trimera* and *Aloe vera*, which showed that the plants grow taller in shading environment [23,24]. The increase in PH under lower light intensity could also be attributed to the production of auxin that is triggered by the stress and, thus, helps to accelerate shoot elongation [25]. Another study on tobacco seedlings [26] also showed the production of taller plants under low irradiance.

However, LN was observed to increase under control, zero shading, which showed the highest LDW under T1 on the 25th day (Figure 2). The results suggested that the increase in shading levels would decrease the number of leaves that were produced in plants, as plants may be focusing more on enlarging the size of the leaves by cell multiplication, instead of producing more leaves in order to be able to absorb sunlight more efficiently [27,28]. A high LN under T1 indicates that zero shading may improve the leaves biomass of *P. minus*, and these results are aligned with the findings that were recorded in common sage (*Salvia officinalis* L.), which showed that sage plants produced a greater number of leaves and exhibited higher productivity under high light intensity [29].

Besides different shading levels that were subjected onto the plants, the duration of exposure also showed significant results on the biomass of *P. minus*. An increasing trend could be observed between dry weights of plants from time interval of eight weeks and 16 weeks, even under low light intensity. This suggests the adaptability of *P. minus* towards low light intensity, as the plants managed to strive in terms of growth. Nevertheless, low LDW can be observed under shaded conditions T2 and T3, which suggests that low light intensities limit the leaves growth of *P. minus*. The highest SDW obtained under T1 and lower RDW that were obtained under T2 and T3 were parallel to the results obtained in a study on *Aloe vera*, which obtained twice higher dry mass of shoot under full sunlight and concluded that the reduced dry mass of root was caused by a restriction of carbon allocations to roots under low irradiance [23]. In addition, low light intensity will also cause a limitation in the supply of carbohydrates from the photosynthesis process, which will affect the roots more when compared to the shoots, as roots depend on the shoots for carbohydrate [30]. RSR was not significantly influenced by the different shading levels, which aligned with the study that was reported by Jose et al., 2002 [31] where light exerted no significant influence on the root:shoot ratio, highlighting that root:shoot ratio changes were commonly found under nutrient stress. Nevertheless, favourable condition for plants in many aspects, such as the soil water content, surrounding temperature, good irrigation, fertilization practices, and the light intensity level around the plants, may result in a reduced root-shoot ratio [32]. The allocation of plant parts biomass between leaves, shoots, and roots depends on species, ontogeny, and on the environment around the plants [33]. There was no fixed pattern observed for this allocation in *P. minus* under light stress from this study. Strong positive correlation can be seen between SDW and RDW (r^2^ = 0.998; *p* ≤ 0.01) (Table 2). High interaction between LDW and SDW (r^2^ = 0.988; *p* ≤ 0.01) and between LDW and RDW (r^2^ = 0.985; *p* ≤ 0.01) can also be observed from Table 2. These correlations suggest that, as LDW increase, the SDW and RDW will also increase. Changes in the allocation of dry mass between roots and shoots may enable plants under stress conditions to be able to obtain enough absorption of water and nutrient [34]. A balanced growth of plants hypothesis suggests that the plants favour more in allocating biomass to the organ that is harvesting the resources, such as leaves and roots, to increase resource capture following the imbalance of carbon fixation and soil nutrient gain, as the environment condition and resources availability will often fluctuate in nature [35].

The significant influence of the different shade levels on *P. minus* exhibit the highest photosynthetic rate under intermediate light intensity T2 (Table 1). Plants under high irradiance would commonly have higher photosynthesis rate when compared to plants under the shade [33]. However, a previous study on *Salvia officinalis* reported that long exposure of plants under high light intensity might damage the photosynthesis apparatus, which leads to its partial loss of the photosynthetic function [29]. A study on *Eugenia uniflora* L. also reported that plant leaves showed lower photosynthetic pigment contents under high light intensity as compared to low light intensity, although the chlorophyll pigment will usually be synthesised and photo-oxidised under the presence of light, but excessive light may cause great degradation in the green pigmentation [36]. Plants that are grown under low light intensity are known to optimise the photosynthesis process efficiently by increasing the pigment density per unit leaf area [37]. The present study showed that the highest photosynthesis rate of *P. minus* was under T2, being directly proportional to the leaf pigment contents, which were also found to be the highest under T2 (Table 1). A study on safflower [38] also found that a higher photosynthesis rate in the subject was accompanied by a higher content in chlorophyll. A strong positive correlation can be observed between the photosynthesis rate and leaf pigment, Chl a (r^2^ = 0.536, *p* ≤ 0.01), Chl b (r^2^ = 0.540, *p* ≤ 0.01) and Chl a+b (r^2^ = 0.546, *p* ≤ 0.01) (Table 2). It is also more useful to express photosynthesis relative to leaf chlorophyll, as it could reflect the balance in the investment between the capture and utilisation of light [39]. In-situ chlorophyll was the highest under T2 (Table 2), being parallel to the results that were obtained for chlorophyll a and chlorophyll b contents, which were also the highest under T2.

Carotenoids are one of plant synthesised secondary metabolites that act as important radical scavengers [40]. This study showed that 50% shading level under T2 significantly enhanced the production of carotenoid in the leaves of *P. minus* along with the enhancement of chlorophyll content (Table 1), and this observation was supported by strong correlation found between the Car with Chl a (r^2^ = 0.963; *p* ≤ 0.01) and Chl b (r^2^ = 0.946; *p* ≤ 0.01) (Table 2). Similar findings were observed in a study conducted by Bohne and Linden 2002 [41], which showed the highest chlorophyll and carotenoid content were produced when *Chlamydomonas reinhardtii* was grown under low light conditions. The previous study emphasized that the increase in carotenoid may not only depend on the expression of phytoene synthase, but also on the biosynthesis of chlorophyll. The chlorophyll a/b ratio of *P. minus* was not significantly influenced by different shading levels. A decrease in chlorophyll a/b ratio indicates an increase in the amount of chlorophyll b, which is exclusively found in the pigment antenna system of PSII. Thus, the reduced amount of chlorophyll a/b ratio could be due to the enlargement of the antenna system of PSII over time, which positively correlates with the light-harvesting chlorophyll–protein complex (LCHII) [42,43].

Different shading levels also significantly influence the leaf temperature, transpiration rate, and stomatal conductance of *P. minus*. Low leaf temperature under control, T1 as compared to other shading levels is due to the effect of evaporative leaf cooling that will cause a reduction in leaf temperature, as the temperature of the surrounding will indirectly increase under high light intensity [44]. Leaf temperature has also been found to be closely related to many other physiological aspects of plants, especially on the photosynthesis rate and accumulation of pigments [45]. Hence, a high significant positive correlation can be observed between leaf temperature and in- situ chlorophyll (r^2^ = 0.850, *p* ≤ 0.01) (Table 2). In-situ chlorophyll influences the leaf temperature by determining the amount of sunlight absorbed that will further excite the photons, creating energy for the assimilation process and producing heat within the plant [46]. The values of E and Gs were both significantly influenced by the different shading levels and strongly correlated with each other (r^2^ = 0.972; *p* ≤ 0.01), as the degree of the opening and closing of the stomata will control the rate of transpiration. This strong correlation also suggests that the transpiration rate in this study was strongly influenced by stomatal regulation. The closing and opening of stomata at the same time depends on the turgor pressure of the guard cells, which could easily be influenced by light changes [47]. Plants under control, T1 exhibits a low E due to the loss of turgidity in leaves that is caused by the drier air that resulted by the high light intensity, thus reducing the transpiration rate as the stomata close [48]. Low light intensity may lead to a higher relative humidity in the surrounding, which caused a decrease in abscisic acid that regulates the stomatal movement [49], contributing to the malfunctioning of the stomata [50]. This result is parallel with the value of low Gs obtained under T3, and this value was not significantly different with the value of Gs under T1. There were strong correlations between transpiration rate with Chl a (r^2^ = 0.653; *p* ≤ 0.01), Chl b (r^2^ = 0.553; *p* ≤ 0.01), and Chl a+b (r^2^ = 0.61; *p* ≤ 0.05) (Table 2). These correlations seemed to be complex, as the transpiration rate and stomatal conductance are more closely correlated with the process of photosynthesis through the intake of CO_2_ and diffusion of water vapour [51]. We assumed that these significant correlations indicate that the key factor of the increased photosynthesis rate under intermediate light intensity was due to the increased in leaf pigments content, thus affecting the transpiration rate and stomatal conductance that were observed in this study.

Different shading levels as well as duration of treatment exposure were found to be significantly influenced the production of TAC, TPC, and TFC in *P. minus* leaves. *P. minus* leaves that were grown under T3 were found with the highest amount of anthocyanin (TAC) after eight weeks of exposure, but the highest after 16 weeks of exposure show under T2 as compared to other treatments. This result is in contrast to other few studies, which reported that high light intensity induced the production of anthocyanin [52,53]. This is because low light condition is often paired with low photosynthesis, which restricts the production of carbohydrates that play a significant role in anthocyanin biosynthesis [54]. However, such a condition was not observed in this study. These show that *P.minus* under intermediate light intensity is able to give a high photosynthesis rate, thus increasing the anthocyanin synthesis. A strong correlation was observed between TAC with carotenoid content in this study, where r^2^= 0.549, *p* ≤ 0.05 (Table 2). The biosynthetic pathways of both carotenoid and anthocyanin were well established; however, these two pathways were not directly inter-related. Nevertheless, these pathways may be overlapping at the level of induction in response to various stimuli [55], which, in this study, is the light intensity.

The productions of TPC and TFC in the leaves of *P. minus* are the highest under T2 for samples harvested after 8 weeks of treatment exposure. In contrast, after 16 weeks of treatment exposure, 70% shading under T3 was observed to yield the highest amount of TPC and TFC in *P. minus* leaves, being much higher than the TPC and TFC recorded after eight weeks of treatment. The higher production of TPC and TFC under low light condition is aligned with the results obtained in *Orthosiphon stimaneus* [56], which showed that the increase may be due to the increase in the availability of phenylalanine enzyme that ushers the production of carbon-based secondary metabolites, including phenolic and flavonoid compounds. The increase of the photosynthesis rate under T2 due to the increase in-situ chlorophyll content shows the increase of the production of secondary metabolites that are derived from the photosynthetically-produced carbohydrate under the shikimic acid pathway [18,57], as high in-situ chlorophyll indicates a greater amount of sunlight absorption that will produce a higher photosynthesis process, annotated by high correlation between TPC and TFC with in-situ chlorophyll, SPAD (r^2^ = 0.744, *p* ≤ 0.01 and r^2^ = 0.644; *p* <0.05 (Table 2), respectively. The production and accumulation of carbon-based secondary metabolites show significantly correlated with the in-situ chlorophyll content, indicating that the in-situ chlorophyll that is capable of predicting the production of phenolic and flavonoid compounds. High significant correlations are also observed between TPC and TFC with Tleaves, with r^2^ values of 0.798, *p* ≤ 0.01 and 0. 693, *p* ≤ 0.05. We assumed the correlations with Tleaves were highly affected by the values of SPAD, since they were also strongly correlated (Table 2). Meanwhile, a strong correlation between TPC and TFC with a r^2^ value of 0.889, *p* ≤ 0.001 shows that, as the TPC increases, the value of TFC will also increase and this will improve the medicinal value of *P. minus.* The presence of flavonoid is crucial in indicating the biological activity for anti-inflammatory, antiallergic, antiviral, anticarcinogenic activities, and the most important for antioxidant activity due to its ability to reduce free radical formation and scavenge free radical [58]. Several studies reported that phytocompounds found in *P. minus*, such as phenolic and flavonoid, contribute the most to antioxidative and anti-inflammatory activities [59]. In comparison with other medical plants, *P. minus* had been reported to obtain high phenolic content compared to *Curcuma longa* and *Zingiber officinale* [6]. Therefore, the current study suggested *Polygonum minus* to be able to enhance its medicinal properties through shading. Antioxidant agents are believed to be able to prevent carcinogenesis and atherogenesis responsible for cancer and cardiovascular diseases, by passively interfering with oxidative damage to DNA and lipoproteins [60]. Existing data on the usage of antioxidant compounds and chemotherapy showed that antioxidant supplementation led to an improvement in treatment outcomes, increased survival times, higher anti-tumour response, and reduced toxicity [61]. The longer exposure of 16 weeks under a shading level of 50% (T2) has been seen to significantly improve the antioxidant properties of *P. minus* as compared to eight weeks. Shading have been reported to improve antioxidant properties in *Coffea arabica* [62] and *Zingiber officinale* [17], but in contrast to what have been reported on *Labisia pumila* [63] and on the greenhouse-grown lettuce [64]. Based on this research, antioxidant activity was highly affected by the level of Chl a, Chl b, Car, and TAC instead of the values of TPC and TFC due to high correlation values that are shown in Table 2. This shows that the antioxidant properties in *P. minus* are not mainly contributed by the production of TPC and TFC, which aligned with the result that was reported by Mahmud et al., 2019 [65]. Carotenoids and anthocyanin have both long been known as efficient antioxidants, in addition of anthocyanin compound being part of the flavonoid group [66,67,68]. A negative significant correlation between FRAP and DPPH (Table 2) assay shows, that as the value of FRAP reducing power increases, a lesser concentration of *P. minus* extract is needed to inhibit 50% of DPPH radical species.

## 4. Materials and Methods

### 4.1. Treatments Description and Maintanance

The study was carried out in Rimba Ilmu Botanical Garden greenhouse, University of Malaya. *P. minus* plants were propagated for two weeks before being transplanted to media; polyethylene bags that were filled with soilless mixture of burnt rice husk and coco peat in the ratio of 3:1, respectively. Three treatments of shading levels: 0% shading (Control, T1), 50% shading (T2), and 70% shading (T3) were arranged in a Randomized Complete Block Design with 3 shading levels × 4 blocks × 8 replicates. The percentage of shade levels are based on the density of the nylon mesh of the commercial shade cloth. The manufacturers provided this information. The shade levels (T2 and T3) were prepared in rows where the shade levels were represented by black nylon netting canvas with different mesh percentage. The canvas was installed from wall to wall of the greenhouse, forming horizontal shading above the plants in each treatment row. Inner walls were also installed around the shaded compartments, to make sure that all plants were covered. The light intensities were continuously monitored by using a LI-COR LI-250A light meter to ensure the significant range of differences between treatments. The average light intensity (µmol m^−2^ s^−1^) measured under different shading levels during experiment showed that (T1) 0% shading, the control recorded 80.024a ± 6.476, (T2) 50% shading recorded 29.927b ± 4.859, and (T3) 70% shading recorded 11.698c ± 1.018. All of the plants were watered sufficiently every morning and fertilised using NPK green fertilizer (NPK ratio: 10:20:10) once per week.

### 4.2. Plant Growth

Plant height (PH) and leaves number (LN) were measured every two days until the 25th day after transplant. The plant height measurements were taken on the main stem from the reference point on the soil surface towards the tip of the stem by using a measuring tape with an accuracy of ±1 mm. The number of leaves was recorded in total per plant. All of the data were recorded in eight replicates for each block under all treatments.

### 4.3. Biomass Dry Weight and Root-Shoot Ratio

Plants were harvested after a time interval of eight weeks and 16 weeks of planting to measure the leaves dry weight (LDW), shoot dry weight (SDW), and root dry weight (RDW). One harvest time measured for 4 replicates × 4 blocks of all treatments. The harvested plants were divided into different plant parts (leaves, shoots, and roots). All parts were subjected to freeze-drying process using Labconco freeze dryer (Labconco Corporation, MO 64132 USA) at −50 °C. The dry weights of the plant were recorded as the biomass dry weight. The root-shoot ratio was calculated by dividing root dry weight with the above ground dry weight [69].

### 4.4. Leaf Gas Exchange

The measurements of photosynthesis rate (A), leaf temperature (Tleaves), transpiration rate (E), and stomatal conductance (Gs) were obtained by a closed infra-red gas analyser LICOR 6400 Portable Photosynthesis System (IRGA, Licor Inc., Lincoln, NE, USA). The measurements used an optimal condition set of 400 µmol mol^−1^ CO_2_ flux, 30 °C standard cuvette temperature, and PAR of 1200 µmol m^−2^ s^−1^ [70]. Leaf gas exchange measurements were carried out between 10:00 a.m. to 11:00 a.m., on the third fully expanded leaves from the plant apex. The data were recorded in triplicates from each treatment within each block.

### 4.5. In-Situ Chlorophyll Content

In-situ chlorophyll content (SPAD) was recorded using a SPAD-502 meter (Konica Minolta Optic Inc., Tokyo, Japan) once every three days on a mature expanded leaf of each plant. The equipment was calibrated prior to the measurements. The data were recorded in triplicates from each treatment within each block.

### 4.6. Leaf Chlorophyll and Carotenoid Analysis

The chlorophyll a (Chl a), chlorophyll b (Chl b), and carotenoid (Car) were analysed from young, expanded leaves. Approximately 0.1 g leaf sample was crushed in liquid nitrogen by using mortar and pestle. The sample was ground in 10 mL absolute methanol using chilled mortar and pestle before being incubated under −20 °C in the dark for 24 h. The relative chlorophyll and carotenoid levels were measured in triplicates with a spectrophotometer (Thermo Scientific Multiskan GO) at 665.2, 652.4, and 470 nm wavelengths after 10-fold dilution. The pigment concentrations of chlorophyll a, chlorophyll b, and carotenoid were calculated following the methods by Lichtenthaler and Claus, 2001 [42], and then expressed as mg chlorophyll g^−1^ tissue fresh weight (mg g^−1^ FW). Next, the total chlorophyll (Chl a + b) and chlorophyll a and b ratio (Chl a/b) were calculated following Li et al., 2018 [71].

Chl a (µg/mL): 16.72 (A665.2) − 9.16 (A652.4)

Chl b (µg/mL): 34.9 (A652.4) − 15.28 (A665.2)

Car (µg/mL): (1000(A470) − (1.63(Chl a) − 104.96 (Chlb))/221

### 4.7. Total Anthocyanin Content

The extraction process that was used for the total anthocyanin content (TAC) assay was conducted, as previously described by Giusti and Wrolstad, 2001 [72] using pH differential method with some modifications. 0.2 g of freeze dried leaves was ground in 10 mL of absolute methanol. The methanolic extract was separately diluted with two types of buffer: potassium chloride (0.025 M) at pH 1.0 and sodium acetate (0.4 M) at pH 4.5 using the ratio 1:4 (one part test portion and four parts buffer). The absorbance of the samples was measured at 510 and 700 nm. The TAC was calculated while using the following formula:

Total anthocyanin content (mg/L) = Ab × MW × df × 1000 × ε × 1

where

Ab = (A510 − A700) pH1.0 − (A510 − A700) pH4.5

MW = Molecular weight of cyanidin-3-glucosode (449.2 g/mol)

df = the dilution factor

ε = Extraction coefficient (296,000 mol/g)

### 4.8. Total Phenolic and Flavonoid Content

The extraction and quantification for total phenolic and flavonoid contents followed the methods that were described by Yusof et al., 2018 [73]. The leaf samples were harvested over two time intervals (eight weeks and 16 weeks). The methanolic extract of *P. minus* was prepared after the leaf samples were freeze-dried. 0.5 g of freeze dried leaves were soaked and ground in 30 mL of absolute methanol using chilled mortar and pestle. The sample mixtures were then incubated at −20 °C for 24 h before being filtered using filter paper. The filtrates were pooled and then evaporated under 45 °C using rotary evaporator before adjusted to a concentration of 20 mg mL^−1^.

Folin–Ciocalteu reagent (10%) was used to determine the total phenolics content (TPC) of the leaf samples. 0.1 mL of the sample extract was mixed with Folin–Ciocalteu reagent (0.75 mL). 2% aqueous sodium carbonate (0.75 mL) was added into a test tube and then incubated in the dark for 45 min. The blanks were prepared by using absolute methanol and the absorbance was recorded at 765 nm. Standard calibration curve was prepared with a series of gallic acid standards (0.01, 0.02, 0.03, 0.04, 0.05, and 0.06 mg/mL). The results were represented as mg g^−1^ dry weight of leaves (mg g^−1^ DW). For total flavonoid content (TFC), 0.5 mL of the sample was mixed with 1.5 mL of absolute methanol in a test tube that was covered with aluminium foil, and it was left for 5 min. Next, 10% 0.10 mL of AlCl₃ (AlCl₃•6H₂O) was added followed by the addition of 0.1 mL NaOH 1 M and 2.8 mL of distilled water. The absorbance was measured at 415 nm after 40 min. of incubation with quercetin as a standard and the results were expressed as mg g^−1^ dry weight of leaves (mg g^−1^ DW).

### 4.9. Ferric Reducing Antioxidant Power (FRAP)

The FRAP assay was performed based on the method that was described by Benzie & Strain, 1999 [74] with slight modifications. Approximately 300 μL of methanolic plant extract was mixed with 10 μL FRAP reagent and it was incubated in microplate wells at room temperature in the dark for 30 min. The absorbance was recorded at 593 nm. A series of stock solution at 1.0, 2.0, 3.0, 4.0, 5.0, and 6.0 mg/mL of ferrous sulphate (FeSO_4_) were prepared to generate a standard curve (r^2^ = 0.9902). The results obtained were expressed as mg of ferrous sulphate equivalent per gram of dried extract.

### 4.10. DPPH Radical Scavenging Assay

The 2,2-Diphenyl-1-picrylhydrazyl (DPPH) free radical scavenging activity of each sample was determined according to the method that was described by Yusof et al., 2018 [73]. 50 µL of extract at six different concentrations (0.5, 1.0, 2.0, 3.0, 4.0, and 5.0 mg/mL) was added to 150 µL of DPPH solution (60 mM) in each well of a 96-well plate. The change in absorbance at 515 nm was measured after 30 min. incubation in room temperature. The obtained data will then be used to determine the concentration of the sample that is required to scavenge 50% of the DPPH free radicals (IC_50_). The percentage of inhibition was plotted against the concentration and the IC_50_ was obtained from the fitted linear curve. A lower IC_50_ denotes a more potent antioxidant.

### 4.11. Statistical Analysis

All of the data were analysed using the SPSS Processor (Statistical Package for the Social Sciences, Version 25) for descriptive statistics and one-way analysis of variance (ANOVA) with Tukey’s post hoc test at a 5% level of probability in significant difference. The standard error of differences between the means was calculated with the assumption that the data were normally distributed and equally replicated. The association and relationships between parameters are shown using Pearson’s correlation analysis.

## 5. Conclusions

From the study, it was found that control (0% shading level) produced highest plant biomass; however, a 50% shading level show the highest leaf gas exchange properties, leaf pigment contents, and secondary metabolites (TAC, TPC), including antioxidant properties (FRAP). Under 50% shading level, *P. minus* showed adaptation well up to 16 weeks’ exposure. It was found *P. minus* might tolerate low light intensity by an increase in its chlorophyll content, photosynthesis rate, transpiration rate, TAC, TPC, and FRAP, while reducing DPPH, which indicates high antioxidant properties under these conditions.

## Figures and Tables

**Table 1:** The effects of the different shading levels on the leaf gas exchange and leave pigments of *P. minus* (data are means of treatments, *N* = 48; Rep = 4; Control, T1 = 0% Shading; T2 = 50% Shading and T3 = 70% Shading; FW = Fresh Weight; Means with different letters on top of each standard error of means are significantly different at *p* ≤ 0.05 between shading treatments).

Treatments		T1	T2	T3
Leaf gas exchange	A (µ mol CO_2_ m^−2^ s^−1^)	6.845 ± 1.68 b	14.892 ± 3.65 a	6.860 ± 2.36 b
	Tleaves (°C)	33.81 ± 0.381 b	36.10 ± 1.061 a	35.69 ± 0.432 a
	E (mmol H_2_O m^−2^ s^−1^)	0.259 ± 0.0008 b	0.3399 ± 0.0008 a	0.282 ± 0.0008 b
	Gs (mol H_2_O m^−2^ s^−1^)	0.006 ± 0.00078 b	0.007 ± 0.00083 a	0.0058 ± 0.000818 b
Pigments	Chl a (mg g^−1^ FW)	2.843 ± 0.128 c	5.251 ± 0.539 a	3.9 ± 0.161 b
	Chl b (mg g^−1^ FW)	3.308 ± 0.031 bc	5.317 ± 0.785 a	3.848 ± 0.338 ab
	Car (mg g^−1^ FW)	1.474 ± 0.052 bc	2.771 ± 0.369 a	2.244 ± 0.195 ab
	Chl a + b (mg g^−1^ FW)	6.151 ± 0.144 bc	10.568 ± 1.324 a	7.748 ± 0.497 ab
	a/b ratio	0.859 ± 0.036 a	1.013 ± 0.058 a	1.029 ± 0.046 a
	SPAD	26.258 ± 0.822 c	33.716 ± 1.198 a	30.305 ± 0.916 b

Note: A: Photosynthesis rate, Tleaves: Leaf temperature, E: Transpiration rate, Gs: Stomatal conductance, Chl a: Chlorophyll a, Chl b: Chorophyll b, Chl a + b: Total chlorophyll content, Car: Carotenoid, a/b ratio: Chlorophyll a and b ratio, SPAD: In-situ chlorophyll.

**Table 2:** Significant Pearson’s correlation between parameters measured in this experiment. The (*) indicates significant difference at *p* ≤ 0.05 while (**) indicates significant different at *p* ≤ 0.01.

		1	2	3	4	5	6	7	8	9	10	11	12	13	14	15	16	17	18	19
1	PH	1	-	-																
2	LN	0.692 **	1	-																
3	LDW	−0.276	0.212	1																
4	SDW	−0.185	0.266	0.988 **	1															
5	RDW	−0.184	0.264	0.985 **	0.998 **	1														
6	RSR	−0.500	0.008	0.793 **	0.746 **	0.764 **	1													
7	A	−0.088	−0.210	−0.040	0.044	0.045	−0.162	1												
8	E	0.075	−0.495	−0.326	−0.300	−0.323	−0.441	0.242	1											
9	Gs	0	−0.530	−0.335	−0.304	−0.326	−0.434	0.313	0.972 **	1										
10	Tleaves	0.484	0.026	−0.209	−0.189	−0.199	−0.344	−0.104	0.501	0.293	1									
11	Chl a	0.342	−0.277	−0.398	−0.314	−0.316	−0.557 *	0.536 *	0.652 *	0.620 *	0.468	1								
12	Chl b	0.145	−0.367	−0.304	−0.254	−0.255	−0.394	0.540 *	0.553 *	0.508	0.416	0.939 **	1							
13	Car	0.311	−0.254	−0.397	−0.329	−0.333	−0.595 *	0.482	0.513	0.477	0.42	0.963 **	0.946 **	1						
14	Chl a+b	0.244	−0.329	−0.355	−0.287	−0.289	−0.480	0.546 *	0.610 *	0.570 *	0.448	0.984 **	0.986 **	0.969 **	1					
15	a/b ratio	0.524	0.262	−0.263	−0.164	−0.168	−0.475	0.033	0.239	0.282	0.105	0.149	−0.196	0.038	−0.030	1				
16	SPAD	0.586 *	0.089	−0.330	−0.321	−0.321	−0.448	−0.195	0.388	0.213	0.850 **	0.463	0.43	0.456	0.453	0.027	1			
17	TAC	−0.094	−0.410	−0.382	−0.330	−0.331	−0.552	0.483	0.309	0.434	−0.255	0.522	0.413	0.549 *	0.473	0.351	−0.103	1		
18	TPC	0.805 **	0.361	−0.281	−0.204	−0.212	−0.553 *	0.072	0.357	0.212	0.798 **	0.529	0.39	0.498	0.464	0.36	0.744 **	−0.119	1	
19	TFC	0.845 **	0.498	−0.428	−0.365	−0.378	−0.680 *	−0.162	0.154	0.029	0.663 *	0.353	0.192	0.387	0.274	0.445	0.644 *	−0.085	0.889 **	1

Note: PH: Plant height, LN: Leaves number, LDW: Leaves dry weight, SDW: Shoot dry weight, RDW: Root dry weight, RSR: Root-shoot ratio, A: Photosynthesis rate, E: Transpiration rate, Gs: Stomatal conductance, Tleaves: Leaf temperature, Chl a: Chlorophyll a, Chl b: Chlorophyll b, Car: Carotenoid, Chl a+b: Total chlorophyll a and b, a/b ratio: chlorophyll a and b ratio, SPAD: In-situ chlorophyll, TAC: Total anthocyanin content, TPC: Total phenolic cotent, TFC: Total flavonoid content.

**Table 3:** The effects of the different shade levels on antioxidant activities of *P. minus* extract. (Data are means of treatments, *N* = 36; Rep = 3; Control, T1 = 0% Shading; T2 = 50% Shading; and, T3 = 70% Shading; DE: Dry Extract; means with different letters on top of each standard error of means are significantly different at *p* ≤ 0.05 between shade levels).

Treatments	FRAP (mg g^−1^ DE)		DPPH IC_50_ (mg mL^−1^)	
	8 Weeks	16 Weeks	8 Weeks	16 Weeks
T1	2.041 ± 0.004 ab	1.930 ± 0.404 ab	3.338 ± 0.001 a	4.202 ± 0.423 a
T2	1.933 ± 0.016 ab	2.616 ± 0.071 a	2.543 ± 0.001 ab	0.657 ± 0.007 b
T3	1.641 ± 0.019 b	1.457 ± 0.036 bc	2.168 ± 0.340 ab	1.949 ± 0.571 ab

**Table 4:** Significant Pearson’s correlation between secondary metabolites and antioxidant activities. The (*) indicates significant difference at *p* ≤ 0.05, while (**) indicates significant different at *p* ≤ 0.01.

		1	2	3	4	5	6	7	8
1	Chl a	1							
2	Chl b	0.939 **	1						
3	Car	0.963 **	0.946 **	1					
4	TAC	0.522	0.413	0.549 *	1				
5	TPC	0.529	0.390	0.498	−0.119	1			
6	TFC	0.353	0.192	0.387	−0.085	0.889 **	1		
7	FRAP	0.734 **	0.598 *	0.707 **	0.859 **	0.246	0.185	1	
8	DPPH	−0.209	−0.110	−0.330	−0.666 *	−0.034	−0.202	−0.590 *	1

Chl a: Chlorophyll a, Chl b: Chlorophyll b, Car: Carotenoid, TAC: Total anthocyanin content, TPC: Total phenolic content, TFC: Total flavonoid content, FRAP: Ferric Reducing Antioxidant Power, DPPH: 2,2-diphenyl-1-picryl-hydrazyl-hydrate.
